# To Reveal or Not to Reveal? Observation of Social Outcomes Facilitates Reward Processing

**DOI:** 10.3389/fnins.2020.579702

**Published:** 2021-01-11

**Authors:** Qiang Shen, Lian Zhu, Liang Meng, Wenwei Qiu, Qingguo Ma, Richard P. Ebstein, Jia Jin

**Affiliations:** ^1^School of Management, Zhejiang University of Technology, Hangzhou, China; ^2^School of Journalism and Communication, Shanghai International Studies University, Shanghai, China; ^3^Laboratory of Applied Brain and Cognitive Sciences, School of Business and Management, Shanghai International Studies University, Shanghai, China; ^4^College of Economics and Management, China Jiliang University, Hangzhou, China; ^5^School of Management, Zhejiang University, Hangzhou, China; ^6^Academy of Neuroeconomics and Neuromanagement, Business School, Ningbo University, Ningbo, China

**Keywords:** intrinsic motivation, social feedback, event-related potential, feedback-related negativity, reward processing

## Abstract

Motivation is a key topic that comprises considerable theoretical and practical implications, and its study is gaining increasing traction in recent years. Employing both behavioral and neural techniques, previous studies examined the extent to which intrinsic and extrinsic motivations collectively shape individual decision making. Investigations found that both processes play indispensable and interactive roles in choice behavior. However, despite its importance, little is known respecting the role of extrinsic social factors in contributing to individual variations in intrinsic motivation. Toward elucidating the role of extrinsic social factors in motivated decision making, the current study implements the stop watch task, combined with hyper-recording electrophysiological measurements. With the electrophysiological toolkit, our goal is to bring to light how extrinsic social signals impact intrinsic motivation and shape the reward processing over success and failure at the succeeding stage. Empirically, we show that, following social outcome presentation, there is an increased divergent feedback-related negativity (FRN), which reflects the failure/success discrepancy at the outcome stage of choice behavior. In summary, this study demonstrates the saliency of social information in intrinsic motivational processes that underpin success-failure outcomes.

## Introduction

By nature, we human beings are social animals. To conform to the social norm, maintain social image, or seek social status, individual behavior is not only guided by primary or secondary rewards, yet also shows sensitivity to context, especially the social milieu. Previous studies have reported that goal-directed behaviors such as financial outcomes involving monetary gains, as well as exposure to social feedback, elicit the activation of reward-related brain regions including the nucleus accumbens, suggesting the existence of a common brain currency for financial and social reward (Spreckelmeyer et al., 2009). Interestingly, behavioral and recent neuroimaging studies suggest that offering a monetary reward eliminates intrinsic motivation, and narrows the discrepancy between success and failure response toward the subsequent outcome (Deci, 1971; Murayama et al., 2010; Ma et al., 2014). However, it remains unclear whether social feedback, like financial reward, also modulates intrinsic motivation and shapes the reward processing at the stage of outcome evaluation.

Self-determination theory (SDT) is a highly cited milestone toward understanding the mechanisms mediating the psychological construct of motivation (Deci and Ryan, 2012). SDT makes an important distinction between intrinsic and extrinsic motivation. Hence, motivation can be characterized not only by its strength or level of intensity but also by its orientation, intrinsic or extrinsic (Weibel et al., 2014). It is important to recognize the saliency not only of the two kinds of motivation but also crucial to understand the interaction between extrinsic and intrinsic motivation and how the one process is sensitive to the other process. In the framework of SDT, the reference point for extrinsic motivation is linked to the basic needs of intrinsic motivation (Weibel et al., 2014). Echoing this insight, numerous studies examined the effects of multiple contextual factors on motivation and how valenced reward and social feedback impacts intrinsic motivation by modulating the three basic needs in SDT, e.g., autonomy, competency, and relatedness (Deci and Ryan, 2000; Abuhamdeh et al., 2014; Gagné, 2014).

To account for the effects of extrinsic motivation on intrinsic motivation, Deci and Ryan (1985) put forward a cognitive evaluation theory (CET) lodged within the overall scheme of SDT (Deci and Ryan, 1985). Empirically, experiments showed that external reward, provided as a performance contingent compensation, reduces intrinsic motivation. CET asserted that the external reward presents an externally perceived locus of causality, which thwarts an individual’s autonomy, one of the three basic inherent psychological needs thereby minimizing intrinsic motivation (Deci and Ryan, 1985; Weibel et al., 2014). Similarly, a social information reward such as verbal praise also affects intrinsic motivation (Deci, 1971; Hou et al., 2002). Enhanced intrinsic motivation is observed for such socially valenced signals and was explained that such signals buttress the individual’s competence need. Externally imposed information fosters an internal locus of causality, which enables individuals to explore and maintain a self-determined state (Ryan, 1982). In sum, whereas performance-based monetary reward undermines people’s intrinsic motivation toward a task of inherent fun, social information such as verbal praise facilitates it (Deci, 1971; Deci et al., 1999).

Concomitantly with the rapid advancement of cognitive neuroscience, investigators exploited the neuroscience toolkit to examine the underlying mechanisms characterizing at the neural level the interplay between intrinsic and extrinsic motivation (Murayama et al., 2016; Ryan and Di Domenico, 2016; Di Domenico and Ryan, 2017). For example, Murayama et al. (2010) examined how performance-based monetary reward interacts with intrinsic ones by developing a novel game, the stopwatch (SW) task. This task is inherently interesting to play and elicits intrinsic motivation on a convenient platform suitable for cognitive neuroscience studies. By integrating functional magnetic resonance imaging (fMRI) techniques with a two-stage stopwatch task, they examined the neural dynamics of how extrinsically added, performance-related pecuniary reward interacts with intrinsically derived motivation. In comparison to the control group where no performance-based monetary reward was offered at the initial step, they found a prominently decreased activation of the ventral striatum with respect to positive feedback once the performance-based reward was withdrawn (Murayama et al., 2010).

In a follow-up study, our group (Ma et al., 2014) extended the stopwatch task to a three-stage task and employed event-related potentials (ERPs) to examine the temporal dynamic mechanism of intrinsic motivation, which is characterized by changes in FRN difference, and its interaction with financial reward. Valence (win/loss) elicited a larger FRN amplitude for loss trials than that of win trials. The study revealed that introducing a monetary incentive contingency at the intermediate stage of the experiment prominently reduced FRN difference at the final stage of feedback where monetary reward was removed (Ma et al., 2014). Consistent with the behavioral findings, both studies suggested that the extrinsic reward acts as a negative modulator that undermines intrinsic motivation. Subsequently, Meng and Ma (2015) examined how the SDT need for autonomy modulated intrinsic motivation and found that if an individual is given the opportunity to increase the amplitude of FRN toward gain and loss at the stage of reward receipt, *viz.*, providing an enhanced feeling of autonomy, then there was enhanced intrinsic motivation and related beneficial outcomes (Meng and Ma, 2015; Meng et al., 2016).

Overall, these studies suggest that appropriate tasks and neural approaches are available to track brain responses toward an understanding of the neural mechanisms underpinning the modulating role of extrinsic reward upon intrinsic motivation. External intervention, such as a reward or a regulation, undermines or “crowds out” intrinsic motivation. There are also situations where an external reward or discipline “crowds in” or strengthens intrinsic motivation and serves to improve performance. Moreover, as discussed, social information provided by individuals or groups affect intrinsic motivation. However, such social information has been the subject of considerably fewer studies than other forms of extrinsic rewards, and little is known regarding its neural basis. Therefore, the current study intended to examine the brain mechanisms that contribute to how social information impinges on intrinsic motivation. Specifically, we apply a three-stage experiment with paired subjects, at the first and last stage of the task; the subjects were instructed to play out a standard SW task individually as we explained above. Crucially, at the middle stage of the task, for the treatment group, the subjects are presented with the outcome of self and the outcome of the paired subjects concurrently, which we named as social information treatment. Such a manipulation makes it possible to induce the sense of social comparison and see the extent to which such information could modulate the evaluation of the motivation at the subsequent session (Festinger, 1954). Technically, we intend to investigate how the externally given social information (extrinsic motivation) dynamically interacts with and modulates an individual’s intrinsic motivation within such a context and reflected at the succeeding stage of outcome evaluation over performance-based win and loss.

As reviewed above, we hypothesize that intrinsic motivation can be crowded in by social information, and that could, in turn, elicits the increased representation of the reward processing at the subsequent outcome stage. To manifest that, we infer that there will be an increased deflection of FRN over the gain-loss at the final stage of the task after the social information stage. Therefore, we predict that, as opposed to the control group, there will be a larger discrepancy for the gain loss difference between the first and last session of the experiment.

## Materials and Methods

### Subjects

Thirty-two healthy male graduate and undergraduate students were enrolled from Zhejiang University. They were all native Chinese speakers, aged from 18 to 25 (mean age = 21.56; *SD* = 1.93) and self-reported as right-handedness. All the participants had normal or corrected-to-normal vision, and did not have any history of neurological disorder or mental disease. Participants were randomly assigned into two groups, control group and social group, with 16 participants in each group. Two unacquainted subjects attended the experiment simultaneously each time. Written informed consent was obtained from all participants prior to the commencement of the experiment. This study was conducted in accordance with the Code of Ethics of the World Medical Association (Declaration of Helsinki) and was approved by the Internal Review Board of Zhejiang University Neuromanagement Lab.

### Stimuli

The entire experiment was composed of three separate sessions with each session including two blocks. Each block contained 30 trials and resulted in 180 trials across the entire experiment. Participants were asked to perform a stopwatch task adapted from the Murayama et al. (2010) study. Specifically, during the stopwatch task, a watch started automatically, and the subjects were asked to stop the watch by pressing a button. If the time required to press the button fell within a ±70 ms deviation from the target time of 5 s, the participant was considered to have won the current trial; or alternatively, if their time to press the button exceeded the predetermined target time, they lost the trial. This ±70 ms deviation was determined by a pilot study of 30 volunteers before the formal experiment and the participants succeeded in approximately half of the trials on average. This trial run allowed us to have a relatively balanced number of trials distributed between win and loss conditions, as well as make the task interesting following Murayama et al. (2010)’s study and our own study (Ma et al., 2014). Previous literature indicated that people obtain the greatest sense of achievement for the tasks of intermediate difficulty.

**FIGURE 1:**
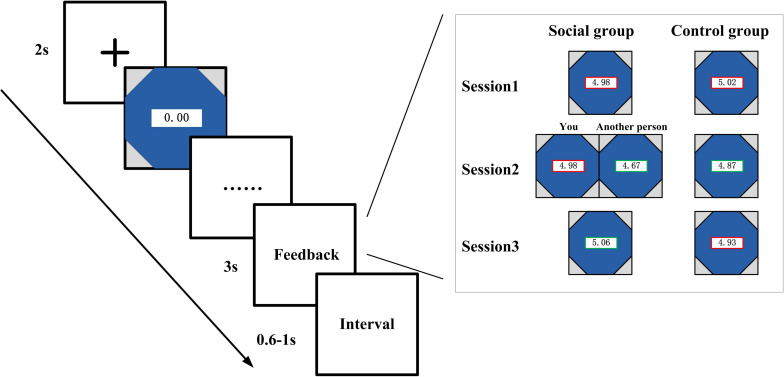
Schematic of the experimental task. As a between-subject design, paired subjects were instructed to play an adapted version of the stopwatch (SW) task. In the treated group, the subjects observed the outcome of paired others in the second session in addition to their own outcome, while the subjects in the control group only see their own performance throughout the whole three sessions.

As shown in Figure 1, in each trial, preceding the presentation of the watch, there was a cross on the screen that lasts for 2 s. The watch stopped once the participants pressed the button, and the elapsed time and performance outcome was revealed on the screen for 3 s. The interval time between trials varied from 600 to 1,000 ms. All the stimuli were presented sequentially in the center of the CRT computer screen (6.2 × 6.2).

Both groups of participants (social and control condition) were instructed to finish three sessions of stopwatch task. For the social condition participants, they were shown the performance outcome of their own and their partner’s in the second session of the experiment. There are four conditions in this situation, e.g., both win, both lose, player wins—companion lose, player lose—companion wins. In the first and third sessions, the social control participants were only shown their own outcomes. The control condition participants were only shown their own performance at the feedback stage throughout all the three sessions. The only difference between control condition and social condition was that the subjects in the social condition were shown the outcome of each other’s performance in the second session of the experiment, whereas participants in the control condition only see their own performance in all the sessions.

### Procedure

The two paired participants were guided to sit 1 m away from a computer-controlled CRT monitor in two separate shielded rooms. Stimuli, recording triggers, and response were presented and recorded using E-Prime 2.0 software package, and self-written script in E-prime was applied to synchronize the presentation of the visual stimuli on two separate computers in a simultaneous manner for paired subjects for the social information session (Psychology Software Tools, Pittsburgh, PA, United States). Subjects in both the social and control conditions were endowed with a fixed 60 Chinese *Yuan* payment for their participation right after they accomplished the whole task, which was unrelated with their performance. The formal experiment started after a five-trial pilot practice.

### EEG Recordings and Analyses

The EEG was recorded (band-pass = 0.05–70 Hz; sampling rate = 500 Hz) with Neuroscan Synamp2 Amplifier (Scan 4.3.1, Neurosoft Labs, Inc. Virginia, United States), using an electrode elastic cap with 64 Ag/AgCl electrodes according to the standard international 10–20 system. A frontal electrode site between FPz and Fz was used for the ground, and the left mastoid was chosen for reference. Electrooculogram (EOG) was recorded from electrodes placed at 10 mm from the lateral canthi of both eyes (horizontal EOG) as well as above and below the left eye (vertical EOG). The EOG artifacts were corrected off-line using the method initially proposed by Semlitsch et al. (1986) for all subjects during preprocessing of the raw EEG data. The experiment started only when the electrode impedances were maintained below 5 kΩ. The data were analyzed by using Neuroscan 4.5. Trials containing amplifier clipping, bursts of electromyography activity, or peak-to-peak deflection exceeding ± 100 μV were excluded from final analysis. Data were transferred to the average of the left and right mastoid reference offline. ERPs were digitally filtered with a low-pass filter at 30 Hz (24 dB/octave).

The EEG recordings were segmented for the epoch from 200 ms before the onset of target to 800 ms after the onset, with the first pre-targets of 200 ms as the baseline. As mentioned in the introduction, the overall aim of the current study was to investigate the impact of extrinsic social information on intrinsic motivation. Therefore, we mainly focused on the ERP differences between session 1 and session 3 in the social condition compared with that in the control condition. In session 3, the social condition group was expected to be influenced by the social information provided in session 2, *viz.*, whether their partner won or lost. Therefore, in further EEG analysis, the data were collapsed based on the outcome of session 1 and session 3 separately for both groups.

Based on visual observation of grand average waveforms and previous ERP studies on feedback processing (Leng and Zhou, 2010), two ERP components, FRN and P300 were analyzed. For the analysis of FRN, we chose a time range of 180–220 ms and selected nine electrode sites (F1, Fz, F2, FC1, FCz, FC2, C1, Cz, and C2) in frontal and central areas according to the scalp distribution of FRN and the previous studies about FRN (Yeung and Sanfey, 2004; Jin et al., 2017). Mixed design four-way ANOVA 2 (subject group: control vs. social reward) × 2 (session: 1 vs. 3) × 2 (performance: win vs. loss) × 9 (electrode: F1, Fz, F2, FC1, FCz, FC2, C1, Cz, and C2) was conducted to examine the effect of FRN amplitude of session 1 and session 3 between groups.

For the analysis of P300, six electrodes (CP1, CPz, CP2, P1, Pz, and P2) in the central and parietal areas were selected. A similar mixed design four-way ANOVA was also conducted for P300 analysis between groups in a time window of 250–350 ms. Simple effect analysis was conducted when there was any significant interaction effect among factors. The Greenhouse–Geisser (Greenhouse and Geisser, 1959) correction was applied in all statistical analyses where appropriate.

## Results

### Behavioral Result

**FIGURE 2:**
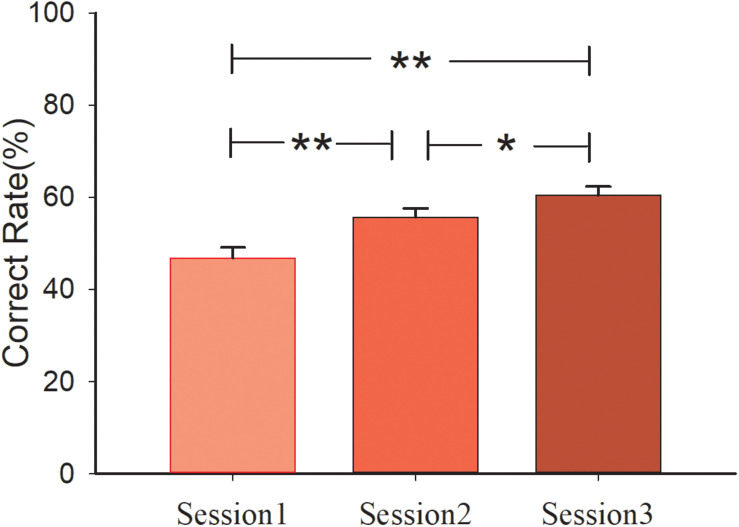
Behavioral results for the three sessions. Participants’ correct rate in three sessions.

A 3 (sessions) × 2 (conditions) mixed design ANOVA analysis of performance (correct rate or win/lose) was conducted with Bonferroni correction. The main effect of session was significant [*F*_(2, 60)_ = 26.496, *p* < 0.001, η^2^ = 0.469]. Pair-wised *t*-test showed that performance of the subjects in session 3 (*M_session3_* = 60.47%, *S.E.* = 0.019) was better than that in session 2 (*p* = 0.018, *M_session2_* = 55.73%, *S.E.* = 0.019). Similar results were observed in session 1 compared with session 3 (*p* < 0.001, *M_session1_* = 46.88%, *S.E.* = 0.023). Performance in session 2 was also better than in session 1 (*p* = 0.002). However, the main effect of condition [*F*_(1, 30)_ < 1] and interaction effect of session and condition [*F*_(2, 60)_ < 1] was not significant. The mean correct rate of the three sessions in the control group are 47.50, 57.29, 61.88%, and in the social group are 46.25, 54.17, 59.06%, respectively. The behavioral results are shown in Figure 2.

### Event Related Potential Result

#### Feedback-Related Negativity Analysis

**FIGURE 3:**
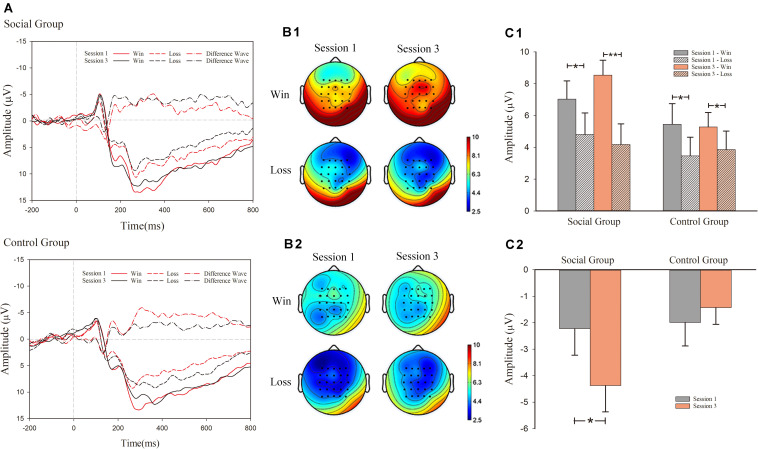
Event-related potential (ERP) results for feedback-related negativity (FRN). **(A)** grand-average ERP waveforms of FRN from the average voltage of all the electrodes were plotted as a function of session (first and third) and outcome (success and failure) in the social and control groups. In addition to that, the differentiated FRN was also plotted. **(B1)** FRN topography map of social group; **(B2)** FRN topography map of control group; **(C1)** mean voltage of FRN in social and control groups; **(C2)** mean voltage of d-FRN in social and control groups.

As shown in Figure 3, mixed design ANOVA results of FRN revealed significant main effects of outcome valence (win or lose) [*F*_(1, 30)_ = 20.339, *p* < 0.001, η^2^ = 0.404], as well as an interaction effect among session, valence (win/lose), and condition (social information provided or not) [*F*_(1, 30)_ = 5.999, *p* = 0.020, η^2^ = 0.167]. However, a main effect of session [*F*_(1, 30)_ < 1, *p* > 0.1] and condition [*F*_(1, 30)_ = 1.265, *p* > 0.1] was not observed.

For the significant interaction effect among the three factors, simple effect analysis was conducted in both groups separately. In the social condition, there was significant main effects of valence (win/lose) [*F*_(1, 15)_ = 14.303, *p* = 0.002, η^2^ = 0.488] and an interaction effect between valence and session [*F*_(1, 15)_ = 6.242, *p* = 0.025, η^2^ = 0.294]. The main effect of session was not significant [*F*_(1, 15)_ < 1]. Similarly, simple effect analysis was also conducted for the significant interaction effect between valence and session. In session 1, the main effect of outcome valence was significant [*F*_(1, 15)_ = 4.956, *p* = 0.042, η^2^ = 0.248]. Loss trials (*M* = 4.809 μV, *S.E*. = 1.355] induced larger FRN amplitude than win trials (*M* = 7.033 μV, *S.E*. = 1.143) (negative polarity: smaller voltage value means larger amplitude). In the after-comparison session 3, a main effect of outcome valence [*F*_(1, 15)_ = 21.429, *p* < 0.001, η^2^ = 0.588] was also observed. Loss trials (*M* = 4.172 μV, *S.E.* = 1.304) induced larger FRN amplitude than win trials (*M* = 8.543 μV, *S.E*. = 0.937).

In the control condition, we observed a significant main effect of valence [*F*_(1, 15)_ = 6.186, *p* = 0.025, η^2^ = 0.292]. However, the main effect of session [*F*_(1, 15)_ < 1, *p* > 0.1], and the interaction effect between valence and session [*F*_(1, 15)_ < 1], was not significant. In the control condition, we observed a larger FRN amplitude in loss trials (*M* = 3.661 μV, *S.E.* = 1.088) compared with win trials (*M* = 5.370 μV, *S.E*. = 1.054). The amplitude was not significantly different across sessions.

We also analyzed the FRN effect in the third session. We observed a significant main effect of valence [*F*_(1, 30)_ = 26.041, *p* < 0.001, η^2^ = 0.465] and the interaction effect between valence and session [*F*_(1, 30)_ = 6.711, *p* < 0.015, η^2^ = 0.183]. It showed that there was a larger FRN amplitude in loss trials (*M* = 4.015 μV, *S.E.* = 0.875) compared with win trials (*M* = 6.914 μV, *S.E*. = 0.650). However, the main effect of session [*F*_(1, 30)_ = 1.552, *p* > 0.1] was not significant. Simple effect analysis was conducted in both groups separately. In the social condition, there were significant main effects of valence (win/lose) [*F*_(1, 15)_ = 21.429, *p* < 0.001, η^2^ = 0.588]. It showed that larger FRN amplitude in loss trials (*M* = 4.172 μV, *S.E.* = 1.304) compared with win trials (*M* = 8.543 μV, *S.E*. = 0.937). In the control condition, there was significant main effects of valence (win/lose) [*F*_(1, 15)_ = 5.104, *p* = 0.039, η^2^ = 0.254]. It showed that larger FRN amplitude in loss trials (*M* = 3.858 μV, *S.E.* = 1.166] compared with win trials (*M* = 5.286 μV, *S.E*. = 0.902).

We also analyzed the FRN effect in the first session. We observed a significant main effect of valence [*F*_(1, 30)_ = 9.948, *p* = 0.004, η^2^ = 0.249]. It showed a larger FRN amplitude in loss trials (*M* = 4.137 μV, *S.E.* = 0.897) compared with win trials (*M* = 6.244 μV, *S.E*. = 0.864). However, the main effect of session [*F*_(1, 30)_ < 1, *p* > 0.1], and the interaction effect between valence and session [*F*_(1, 30)_ < 1], was not significant.

#### P300 Analysis

**FIGURE 4:**
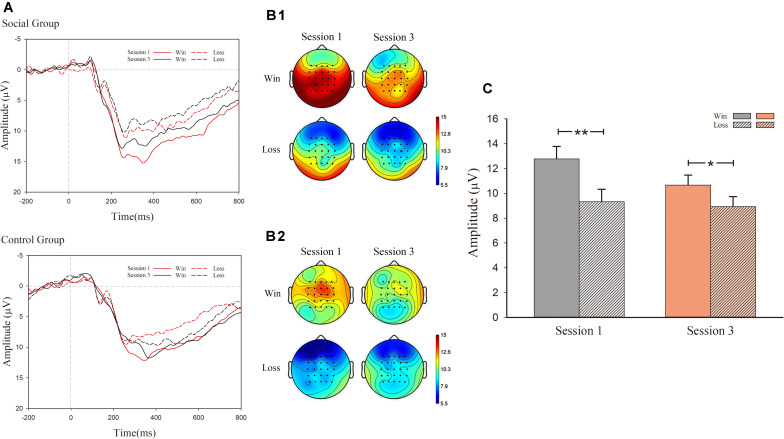
ERP results for P300. **(A)** Grand-average ERP waveforms of P300 from the average voltage of all the electrodes were plotted as a function of session (first and third) and outcome (success and failure); **(B1)** P300 topography map of the social group; **(B2)** P300 topography map of the control group; **(C)** mean voltage of P300 amplitude.

In the analysis of P300, as exhibited in Figure 4, there was a notable main effect for valence [*F*_(1, 30)_ = 22.379, *p* < 0.001, η^2^ = 0.427) as well as an interaction effect between session and valence [*F*_(1, 30)_ = 5.056, *p* = 0.032, η^2^ = 0.144]. However, there were no significant effect main effects of session [*F*_(1, 30)_ = 3.459, *p* = 0.073, η^2^ = 0.103] and condition [*F*_(1, 30)_ = 1.391, *p* > 0.1]. Nor were there any significant effects of interaction for session, valence, and condition [*F*_(1, 30)_ < 1, *p* > 0.1]. For the significant interaction effect between session and valence, simple effect analysis was conducted. In session 1, the main effect of condition was not significant [*F*_(1, 30)_ = 1.534, *p* > 0.1, η^2^ = 0.049], whereas the main effect of valence was significant [*F*_(1, 30)_ = 28.354, *p* < 0.001, η^2^ = 0.486). This observation indicated that outcome characterized by successful hits (*M* = 12.783 μV, *S.E.* = 1.000) displayed a larger P300 amplitude than observed for failed hits (*M* = 9.324 μV, *S.E.* = 1.003). In session 3, the main effect of condition [*F*_(1, 30)_ < 1, *p* > 0.1) was not significant, whereas the main effect of valence was observed [*F*_(1, 30)_ = 6.301, *p* = 0.018, η^2^ = 0.174]. The outcome of successful hits (*M* = 10.671 μV, *S.E.* = 0.798) had larger P300 amplitudes than that of failed ones (*M* = 8.940 μV, *S.E.* = 0.801).

## Discussion

Applying an adapted version of the SW task, the primary aim of the current study was to explore how the social information shape the intrinsic motivation and which sequentially affect the reward processing over performance contingent win loss at the stage of outcome. The current study applies a three-stage version of the SW task. At the intermediate stage, we introduce the *social information*, the simultaneous presentation of the performance contingent outcome of both self and the paired other. The result indicates that, opposed to the control group, after the social information presentation, there is an increased gain loss difference of FRN at the stage of outcome at the last session of the experiment. Nevertheless, this effect was not observed for the subsequent ERPs component P300.

Previous electrophysiological studies showed that FRN was a key candidate ERP component related to motivational and affective evaluation of feedback in various tasks (Nieuwenhuis et al., 2004; Yeung et al., 2004; Ma et al., 2014; Jin et al., 2017). For example, Gehring and Willoughby (2002) investigated the brain processing of monetary gains and losses at the feedback stage, in which they found that prominent larger FRN amplitude was induced by loss feedback compared with the gain one. This is replicated and extended in the subsequent studies (Yeung and Sanfey, 2004; Wu and Zhou, 2009; Ma et al., 2011; Shen et al., 2013). Notably, the gain loss discrepancy of FRN of the electrophysiological toolkit is also observed for the social outcome (Ma et al., 2011; Shen et al., 2013). For example, Fukushima and Hiraki (2009) observed that the FRN is not only responsive to one’s own financial gains and losses, but also to the outcome of their paired friend, although not toward agents generated by computer algorithm (Fukushima and Hiraki, 2009). Therefore, the FRN difference between gain loss could be regarded as a good proxy for the motivational significance of the outcome.

However, we need to note that, unlike the studies that it investigate the extent to which the FRN represents the outcome of others in a different social context, different degrees of social distances or social intimacy, the current study only engages the subjects into social contexts at the middle session and checks how it modulates the amplitude of the FRN at the subsequent session where the outcome is only the result from the executed task, without additional incentive or punishment. In the current study, we observed that, at the last stage, the gain loss difference is not only increased as opposed to control group, but it is also more prominent than the first stage. Therefore, given the motivational significance of FRN, we infer that it might suggest that the social information might increase intrinsic motivation at the stage of the SW task execution. As a result, given the increased motivation toward the stopwatch task, it naturally leads to an increased deflection of the FRN at the subsequent stage of performance-based success failure evaluation. Therefore, the current study extends the previous efforts of the role the FRN under social decision making and indicates the social information could also efficiently modulate subjects’ response to their own performance contingent outcome, not merely responsive to the revealed outcome of others.

In addition, it is also natural and vital to answer why social information could facilitate rather than undermine, as achieved by the extra imposed monetary reward, the intrinsic motivation of the subjects. For example, recently, Meng et al. (2016) developed a tournament version of the stopwatch task and observed that overperformance rather than underperformance of the paired subjects could increase their motivation to play, which was represented by an ERP component called stimulus preceding negativity (SPN) (Meng et al., 2016). To test these potential confounds of social advantage/disadvantage, we also check the paired subjects’ behavioral performance in the middle session. In general, the success rate is similar across treated and controlled groups. Therefore, the increased motivation can hardly be due to either advantage or disadvantage of the performance at the middle stage.

As illustrated in the framework of SDT, external control and self-regulation features of the scenarios could lead to the opposite effect over the intrinsic motivation. The external control could crowd out, while the later can crowd in the motivation. For example, Deci et al. (1999) have revealed that the extrinsic information, which only contains information meaning, could enhance intrinsic motivation toward the task. On the contrary, when it contains control meaning, the extrinsic information would undermine the intrinsic motivation (Deci et al., 1999). Notably, Pittman and D’Agostino (1985) compared the dissociated effect of informational and controlling verbal reward on intrinsic motivation. Their result showed that compared with no-reward control group, informational verbal reward enhanced involvement in task engagement, while the controlling verbal reward did not (Pittman and D’Agostino, 1985). In our current study, different from the direct offer of the financial reward that could result in the imposition of the external control (Murayama et al., 2010; Ma et al., 2014), we only place the information of the other to the player at the second stage. The manipulation neither places extra competition across agents nor offers added financial outcome. Consistent with a previous study, the social manipulation might only contain informational meaning without external control. Therefore, the current study indicates that the non-control social information enhanced self-regulation rather than implementing the social control, facilitating the intrinsic motivation, represented by the increased FRN at the final stage of the task.

For the social information of the counterpart, it can be regarded as a kind of social comparison. Previous studies over this line of research showed that human beings are inclined to evaluate their own opinions and abilities spontaneously; nevertheless, as such a subjective evaluation can hardly be objective or accurate, social comparison is often introduced into the process of self-evaluation in a natural manner (Festinger, 1954). For example, Suls and Wheeler (2000) suggested that an individual’s social comparison motive stems from the pursuit of self-improvement, which motivates themselves to do better (Suls and Wheeler, 2000). Similarly, Wayment and Taylor also indicated that people evaluate their strengths and weaknesses by social comparison and tell themselves to do better for self-improvement, which could lead them to gain satisfaction, competency, and sense of achievement (Wayment and Taylor, 1995). Gervey (2003) assumed that social comparison lead to self-evaluation or self-enhancement, both of which can be attributed to affective motivation (Gervey, 2003). Therefore, in the current study, when the subjects received their counterparts’ performance as social information, they gradually absorb it into the process of self-evaluation. As a result, in the subsequent stage, no matter whether this social information was withdrawn or not, they still perform better for self-improvement, which makes them become more affective to the outcome at the feedback stage in the following session, resulting in increased amplitude of FRN discrepancy.

For the significance of the ERP components at the outcome stage, in addition to FRN, there is also a prominent deflection of ERP component P300. In general, we observed a general stage effect of P300 both for social and control groups. The first stage has larger P300 deflection than that of last. Such findings were in accordance with our previous investigation over the undermining effect of monetary reward to intrinsic motivation, in which we also observe a prominent stage effect of P300 (Ma et al., 2014). This indicates that, as the task proceeded, the subjects’ attention to the outcome was reduced gradually as the time elapses. Furthermore, P300 also showed a pronounced effect for gain–loss discrepancy, and a larger P300 amplitude was induced in the win condition than in the loss condition, which is consistent with the previous findings that the P300 could also reflect the valence of the stimuli in addition to the magnitude of the outcome (Wu and Zhou, 2009). However, dissimilar with the varied effect or gain loss outcome across stage and condition for FRN, there is only the main effect of valence for P300. Therefore, in a task with motivation, it suggests that there is a dissociated role of FRN and P300. The FRN could validly represent the social information effect, while it is absent for P300.

## Conclusion

To sum up, adopting a three-stage version of SW task, this study examined the extent to which social information affect the intrinsic motivation and therefore modulate the reward processing at the stage of outcome evaluation. The current result provides the first electrophysiological evidence, to our knowledge, for the enhancement of social information to intrinsic motivation, which leads to the increased gain loss divergence processing. We suggest two potential explanations for the current findings. One possible channel is that social information, as suggested by the SDT, only conveyed informational meanings but without control implications like monetary reward did; therefore, such informational extrinsic incentive would enhance intrinsic motivation. The alternative account is that social information might have social comparison implication, which prompts the subjects’ drive to perform better for self-improvement.

There are also some limitations to the current study. First, the current only recruited male participants to reduce the heterogeneity of behavioral and electrophysiological responses toward social information across gender. Therefore, it is an open question for further studies to investigate the extent to which social information modulates the motivational effect for female participants and explore the potential gender difference. Second, the current research only presents the results from the temporal processing of ERP waveform rather than the spatial localization. Future studies can carry out fMRI studies or conduct source reconstruction analyses of EEG to examine the potential sources of ERP components over motivational processing. Last but not the least, the current study mainly focuses at the stage of outcome evaluation for the reward processing toward the performance contingent win–loss outcome. The future motivation studies could concentrate at the task execution stage and examine how the modulated factors like the social factors we studied here in the current research modify the inherent processing and pin down the underlying mechanism.

## Data Availability Statement

The data analyzed in this study is subject to the following licenses/restrictions: Data can be sent to the readers if they request to access the data after it is published. Requests to access these datasets should be directed to JJ, jinjia.163@163.com.

## Ethics Statement

The studies involving human participants were reviewed and approved by the Internal Review Board of Zhejiang University Neuromanagement Lab. The patients/participants provided their written informed consent to participate in this study.

## Author Contributions

QS: conceptualization, methodology, and writing-original and revised draft preparation. LZ: conceptualization, methodology, and writing-revised manuscript. LM: conceptualization writing-original preparation. WQ: software and methodology. QM: supervision. RE: conceptualization and writing-revised manuscript. JJ: conceptualization, methodology, data curation, and writing-original and revised draft preparation. All authors contributed to the article and approved the submitted version.

## Conflict of Interest

The authors declare that the research was conducted in the absence of any commercial or financial relationships that could be construed as a potential conflict of interest.
